# Model-based prototype design, establishment and operation of ventilation system for underground gymnasium

**DOI:** 10.1016/j.heliyon.2024.e36055

**Published:** 2024-08-09

**Authors:** Ran Meng, Hui Li, Zhiyong Zhang, Chen Dong

**Affiliations:** a Laboratory of Virtual Reality and Simulation Technology, Shandong Sport University, Jinan, 250102, China; b Shandong Water conservancy vocational college, Rizhao, 276826, China; c Krirk University, Bangkok, 10220, Thailand

**Keywords:** Underground small indoor gymnasiums' ventilation system, Model-based prototype construction, Global parameter sensitivity analysis, Dynamic response optimization, Extended Multiphysics simulation

## Abstract

Underground small indoor gymnasiums (USIG) are important public places, it is vital to design and build a very economical and efficient ventilation system for effective closed-loop regulation of temperature and gases concentration at prescribed levels. In the article, the model-based prototype design, establishment and operation were proposed and applied to closed-loop control system of the underground small indoor gymnasiums’ ventilation system (USIGVS). First of all, the extended Multiphysics model was developed through feedback connecting the 3D Multiphysics model of air flow rate, temperature, O_2_ and CO_2_ concentration with a 0D proportional-integral-derivative (PID) controller via Neumann boundary condition, hence a close-loop USIGVS was constructed for feedback control of temperature and gases concentration in ping-pong USIG. Simultaneously, a cost function sufficiently representing the design requirement was formulated. Then global parameter sensitivity analysis (GPSA) was applied for sensitivity ranking of parameters including geometric parameters of USIGVS and tunable parameters of PID controller. The GPSA proved that sensitivity ordering of the cost function to each parameter was proportional gain (*k*_*p*_) > derivative gain (*k*_*d*_) > distance from left inlet to bottom (*r*) > distance from outlet pipe to bottom (*d*) > integrative gain (*k*_*i*_) > distance from upper inlet pipe to left (*h*), respectively, and the *k*_*p*_, *k*_*d*_ and *r* was the parameter influencing the cost function the most. The optimal parameters determined by both GPSA and response optimization were *k*_*p*_ = 3.17 m^4^ mol^−1^ s^−1^, *k*_*d*_ = 1.49 m^4^ mol^−1^, *r =* 2.04 m, *d =* 3.12 m, *k*_*i*_ = 0.37 m^4^ mol^−1^ s^−2^ and *h* = 3.85 m. Finally, the closed-loop USIGVS prototype with optimal parameters was designed and established through real-time simulation. The real-time operation confirmed that the temperature and gases concentrations were robust maintained at prescribed levels with desired dynamic response characteristics and lower power consumption, and the expected requirements were achieved for the design, establishment and operation of closed-loop USIGVS control system prototype.

## Highlights

• Close-loop USIGVS control system with the optimal parameters obtained.
• Response optimization can maintain the temperature and gases concentrations at prescribed levels.
• This technique can greatly save time and cost in construction of USIGVS prototype.

**Table:** Nomenclature list

**w**	Air flow rate
*ρ*	Air density
*μ*	Air viscosity
T	Temperature
*p*	Air pressure
**I**	Identity matrix
*C*_*p*_	Heat capacity coefficient of air
*k*	Heat conductivity coefficient of air
*v*	O_2_ consumption rate in the human respiratory process
*H*	Respiratory enthalpy
[*O*_*2*_]	O_2_ concentration
*D*_*o*_	Diffusion coefficient of O_2_
[*CO*_*2*_]	CO_2_ concentration
*D*_*c*_	Diffusion coefficient of CO_2_
*R*	Human respiratory quotient
*f*_*r*_	Reference design requirement function
*f*	Current design requirement function
*e*	Difference between *f*_*r*_ and *f*
*k*_*p*_	Proportional gain
*k*_*d*_	Derivative gain
*k*_*i*_	Integrative gain
*r*	Distance from left inlet to bottom
*d*	Distance from outlet pipe to bottom
*h*	Distance from upper inlet pipe to left
*p*_*0*_	Absolute pressure of air
*T*_ext_	External temperature (18 °C) outside the USIG
*α*_*T*_	Weight of temperature in control objective
*α*_*o*_	Weight of O_2_ concentration in control objective
*α*_*c*_	Weight of CO_2_ concentration in control objective
*t*_*f*_	Final time of digital and real-time simulation
*s*	Cost function
**u**	Control law of PID controller
**n**	Normal vector on boundary surfaces
[O_2_]_left_inlet_	O_2_ concentration in left inlet pipe
[CO_2_]_left_inlet_	CO_2_ concentration in left inlet pipe
[O_2_]_top_inlet_	O_2_ concentration in top inlet pipe
[CO_2_]_top_inlet_	O_2_ concentration in top inlet pipe
**v**_in_top_	Air flow rate in top inlet pipe of USIG

## 1 Introduction

Ventilation in underground indoor gymnasium is an important factor in ensuring air circulation and maintaining a comfortable environment [1,2]. An effective ventilation system can provide enough fresh air, remove dirty air, reduce humidity and temperature, and ensure the comfort and health of athletes and spectators [3,4]. The underground small indoor gymnasium (USIG) generally refers to underground court of basketball, football, volleyball, tennis, ping-pong, badminton, billiards and so on. The USIG with small volume and overcrowding often cause temperature and CO_2_ concentration increase as well as O_2_ concentration decrease. If people stay in such environment for long time, it will cause athletes’ premature fatigue, focus loss and inconsistent play, adversely affecting the performance of athletes during the games [5,6]. On the other hand, spectators are usually in a tremendous state of excitement as watching games, the environment of high temperature and CO_2_ concentration as well as low O_2_ concentration can cause them headaches, feelings of dizziness, heart rate increase, blood pressure fluctuation, and even induce sudden cardiovascular diseases of certain audience with underlying diseases [7,8].

Therefore, it is necessary to maintain temperature, O_2_ and CO_2_ concentration in USIG at prescribed levels with desired dynamic response performance. The main solution is currently to design and build a specific underground small indoor gymnasiums’ ventilation system (USIGVS) for regulation of temperature and gases concentration in USIG, respectively. However, there are currently three significant deficiencies in traditional regulation and control method applied for USIGVS, the first is that open-loop regulation without feedback signals [9,10]. For example, the valve of air inflow and outflow pipe was periodically switched on and off according to prescribed frequency and flow rates, venting fresh air with low temperature and high ratio of O_2_ concentration to CO_2_ concentration to USIG, and simultaneously exhausting indoor air with high temperature and low ratio of O_2_ concentration to CO_2_ concentration from USIG. Although the open-loop regulation is extensively used in design, establishment and operation of USIGVS, it is only based on working procedure rather than on-line feedback information in the course of USIGVS operation, hence it is very sensitive to internal variations and external disturbances with low control precision and operating stability [11,12]. The second is that design and building method of USIGVS prototype is based fundamentally on subjective experiences rather than reliable kinetic models, indicating that it will have to take a great deal of time and cost to conduct prototype experiments with trial-and-error methods for realizing prescribed dynamic characteristics of USIGVS [1,13]. The third is that traditional method is decoupling control for temperature and gases concentration in USIG without comprehensively taking constitutive relationships and strong couplings among air flow rate, temperature and gases concentration into consideration, more actuators like exhaust fan and air conditioner put into application indicate more power consumption [14,15].

In order to solve the above three problems systematically, in the research, the model-based design, establishment and operation of USIGVS prototype was put forward and implemented. As is known, ping-pong is the Chinese national ball game, and ping-pong USIG abound in China, therefore USIGVS of ping-pong USIG was selected as research object.

Firstly, the USIGUV was designed as a closed-loop control system by using proportional-integral-derivative (PID) controller to achieve feedback control and regulation of temperature and gases concentration in USIG. An advantage of the closed-loop control system is the fact that the use of feedback makes the system response relatively insensitive to external disturbances and internal variations in system parameters [16]. Secondly, kinetic model of USIGVS was developed based on geometric configuration, governing equations sufficiently taking constitutive relationships and strong couplings among air flow rate, temperature and gases concentration into consideration, and proper boundary conditions, therefore a specific closed-loop USIGVS control system expressed by an extended Multiphysics model was constructed through feedback connection between model of three-dimensional (3D) USIGVS and zero-dimensional (0D) PID controller with Neumann boundary conditions. Thirdly, a cost function sufficiently standing for design requirement was formulated, and the key geometric and tunable parameters were numerically determined and optimized by global parameters sensitivity analysis (GPSA) and response optimization through digital simulation. Finally, the real-time operation results proved that the closed-loop USIGVS control system prototype with satisfied geometric and tunable parameters can effectively regulate the air inflow rate to restore them to the prescribed levels with desired dynamic response performances, as temperature, O_2_ and CO_2_ concentration deviated from their prescribed levels due to respiration and heat release of athletes and audiences.

## 2 Materials and methods

### 2.1 USIG of ping-pong

The selected ping-pong USIG with interior shape was a cuboid of 30m long, 30m wide and 20 m high, a capacity of 1000 spectators and relative humidity 40～80 %, air formaldehyde concentration ≤0.12 mg m^−3^, inhalable particles concentration ≤0.25 mg m^−3^ [17]. The selected USIG was in a garage of a shopping mall with 2.5 m underground and a constant temperature of about 18 °C, located in Jinan, Shandong province, China.

Based on design specification, the nominal states of temperature, O_2_ and CO_2_ concentration in USID was set at 20 °C, 21 %, 0.03 % in order to make athletes and spectators feel comfortable.

### 2.2 Governing equations and boundary conditions

Because of relatively slow flow speed with small Reynolds number (less than 2000) via calculation, thus the Navier-Stokes equation of laminar flow was used as governing equation of air flow in the USIG as follows:

(1) $$\rho \left(T\right)\frac{\partial w}{\partial t}=\nabla \cdot \left[-pI+\mu \left(T\right)\left(\nabla w+\nabla^{T}w\right)\right]$$

where **w** is the air flow rate, *ρ* and *μ* is the air density and viscosity, respectively. Both of *ρ* and *μ* are the function of temperature (T), *p* is the variable of air pressure, **I** is an identity matrix.

Governing equation of temperature is written as follows:

(2) $$\rho \left(T\right)C_{p}\frac{\partial T}{\partial t}+\nabla \cdot \left(-k\nabla T\right)+\rho \left(T\right)C_{p}w\cdot \nabla T=H*v\left(T\right)$$

where *C*_*p*_ and *k* is the heat capacity and conductivity coefficient of air, *v*(*T*) is O_2_ consumption rate in the human respiratory process, and *H* is the respiratory enthalpy, hence the H*v(*T*) represents heat production rate due to human respiration.

Governing equation of O_2_ concentration is written as follows:

(3) $$\frac{\partial \left[O_{2}\right]}{\partial t}+\nabla \cdot \left(-D_{o}\nabla \left[O_{2}\right]\right)+w\cdot \nabla \left[O_{2}\right]=v\left(T\right)$$

where [*O*_*2*_] is O_2_ concentration, *D*_*o*_ is the diffusion coefficient of O_2_.

Similarly, governing equation of CO_2_ concentration is written as follows:

(4) $$\frac{\partial \left[{CO}_{2}\right]}{\partial t}+\nabla \cdot \left(-D_{c}\nabla \left[{CO}_{2}\right]\right)+w\cdot \nabla \left[{CO}_{2}\right]=R*v\left(T\right)$$

where [*CO*_*2*_] is the CO_2_ concentration, *D*_*c*_ is the diffusion coefficient of CO_2_, *R* is human respiratory quotient, hence R*v(*T*) represents CO_2_ production rate due to human respiration. From Eq. (1) ∼ Eq. (4), w, *T*, [*O*_*2*_] and [*CO*_*2*_] were coupled together to formulate a Multiphysics model [18,19].

Furthermore, two types of boundary conditions including *Dirichlet* and *Neumann* conditions were properly specified according to the actual operation of the closed-loop USIGVS control system.

### 2.3 PID controller

The 0 D kinetic model of PID controller is expressed as follows:

$$e\left(t\right)=f_{r}\left(t\right)-f\left(t\right)$$

(5) $$u\left(t\right)=k_{p}e\left(t\right)+k_{i}\int_{0}^{t}e\left(t\right)d\tau +k_{d}\frac{\partial e\left(t\right)}{\partial t}$$

where *f*_*r*_(*t*) and *f*(*t*) is the value of set (reference) and current system output, respectively, and therefore *e*(*t*) is the difference between them. The **u(t)** is the control law of PID controller, *k*_*p*_, *k*_*i*_ and *k*_*d*_ represents proportional, derivative and integrative gain which are tunable parameters, respectively. Based on the extended Multiphysics model of the closed-loop USIGVS control system (Eq. (1) ∼ Eq. (5)), a time-domain design requirement function (DRF) composed of ideal dynamic performance characteristics of temperature, O_2_ and CO_2_ concentration were upfront put forward to sufficiently represent overall performance of closed-loop USIGVS control system, and the strong coupling relationships between temperature, O_2_ and CO_2_ concentration were taken into consideration. Hence, the *f*_*r*_(*t*) and *f*(*t*) respectively represented the reference DRF and current DRF in Eq. (5), and simultaneously *f*(*t*) was used as feedback signals sampled by sensors of temperature, O_2_ and CO_2_ concentration for closed-loop control.

The closed-loop USIGUV control system expressed by an extended Multiphysics model can be constructed through feedback connection between the USIGUV and the PID controller via *Neumann* condition, for robustly maintaining the DRF at nominal level with prescribed spatiotemporal dynamic performance characteristics [20,21]. After the governing equations and boundary conditions were respectively defined in the domains and on the boundaries of USIGVS, the spatiotemporal dynamic characteristics of temperature, O_2_ and CO_2_ concentration can be obtained through computer simulation and finite element analysis (FEA).

### 2.4 GPSA and response optimization

Because kinetic model of air flow rate, temperature and gases concentration in USIG was 3D, while the PID controller was 0D, hence it was difficult to effectively optimize the parameters in kinetic models composed of 3D USIGUV and 0D PID controller for construction of closed-loop USIGUV control system to have satisfied dynamic response performance. So far, the 3D USIG was usually reduced to 0D, namely a variable at a specific point in domain of USIG was kept at a nominal level by PID control and regulation based on feedback signal sampled by sensors [22,23]. However, the variable at a specific point cannot represent overall characteristics of this variable in 3D domain, therefore the ideal control effect with high precision cannot be attained at all. In the article, GPSA and response optimization of the cost function was applied to address this issue.

Traditionally, the sensitivity of the cost function is measured by the partial derivative of the cost function with respect to parameters, which is called local sensitivity analysis. However, this approach can usually be infeasible for complex models like Eq. (1) ∼ Eq. (5), since the closed-loop USIGVS control system was expressed by extended Multiphysics model with uncertain parameters, like current temperature, O_2_ and CO_2_ concentration variations caused by respiration and heat release of athletes and audiences, therefore model do not always have derivatives. Furthermore, local sensitivity analysis is a one-at-a-time (OAT) technique which analyzes the effect of one parameter on the cost function at a time, keeping the other parameters fixed, hence OAT technique can only explore a small fraction of the design space, especially when there are many parameters. Therefore, OAT technique cannot provide insight about how the interactions between parameters influence the cost function. In order to avoid defeats of local sensitivity analysis, in the research, the GPSA was applied for selection of the geometric parameters in USIG and tunable parameters in PID controller which the cost function most sensitive to, the GPSA approach uses a representative (global) set of samples generated by Monte Carlo simulations to explore the design space of parameters. Based on GPSA, further response optimization was performed to obtain the optimal sensitive parameters making the closed-loop USIGVS control system have ideal dynamic response characteristics [24].

### 2.5 Model-based prototype design, establishment and operation

According to the preceding extended Multiphysics model (Eq. (1) ∼ Eq. (5)), the GPSA and response optimization for model-based prototype design, building and operation of the closed-loop USIGUV control system was conducted as follows.

(1) The model parameters were selected and sampled using experimental design principles. For each parameter, multiple values that the parameter can assume were generated. The parameter sample space was defined by specifying probability distributions for each parameter. Random samples are drawn through Monte Carlo simulation from the probability distributions specified for the random parameter generation. The sampling method for *Sobol* quasi-random sequences were applied in the research for highly systematic space-filling from the probability distributions specified for the parameters.
(2) Based on DRF, a cost function was formulated for parameters sensitivity analysis and dynamic response optimization through digital simulation, and the cost function at each combination of parameter values was evaluated using the extended Multiphysics model in combination with digital simulations.
(3) The methods of correlation analysis between the cost function and the samples of parameter included *Pearson* correlation, rank correlation, standardized regression, rank standardized regression, partial correlation and rank partial correlation. The correlation corresponds to the influence of the parameters on the cost function, so the order of parameters the cost function was sensitive to can be obtained correspondingly.
(4) After the parameters the cost function was more sensitive to were obtained, the cost function representing the dynamic response characteristics of the closed-loop USIGVS control system was further optimized by fine tuning these parameters, and the optimum parameters were ultimately determined in the course of minimization of the difference between the actual and reference dynamic response of the closed-loop USIGVS control system.

### 2.6 Software platform for modeling and simulation

The geometric configuration of USIG was drawn by SolidWorks 2018, the Multiphysics model of USIGVS was developed on Comsol Multiphysics 5.4 [25,26], the PID controller was established on Matlab/Simulink [27,28], the joint interface for extended Multiphysics simulation was realized by toolkit of Comsol Multiphysics with Matlab, the GPSA and response optimization was conducted on Matlab2023/Simulink Design Optimization Toolbox, and real-time simulation for model-based design, establishment and operation of closed-loop USIGVS control system prototype was carried out by Matlab2023/Real-Time Workshop [29,30].

## 3 Results and discussion

### 3.1 Extended Multiphysics model derivation

#### 3.1.1 Geometric configuration of USIGVS

**Fig. 1:**
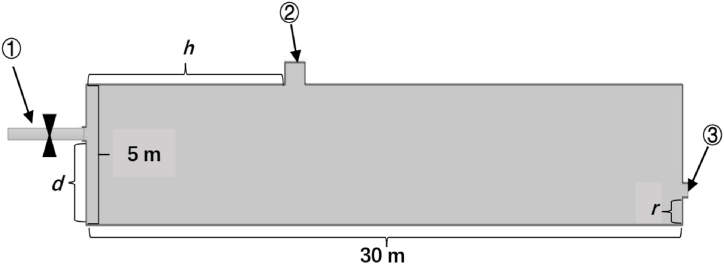
Geometric configuration of USIGVS (①: left inlet pipe with a valve; ②: top inlet pipe; ③: outlet pipe).

The geometric configuration of the predesigned USIGVS (Fig. 1) was a cuboid with symmetric construction. For dramatically reducing computation for Extended Multiphysics simulation, the 3D geometric configuration of USIGVS was reduced to 2D without remarkably diminishing the effectiveness of the calculation and simulation [31].

As illustrated in Fig. 1, there are three undetermined geometric parameters, i.e., distance from upper inlet pipe to left (*h*), and distance from left inlet and outlet pipe to bottom (*d* and *r* respectively) in combination with parameters (*k*_*p*_, *k*_*d*_ and *k*_*i*_) of PID controller need to be specified and optimized. The air flow rate in top inlet pipe of USIG is constant, hence the PID controller regulates the inflow rate (**u**(t)) of the fresh air with low temperature and high ratio of O_2_ concentration to CO_2_ concentration in left inlet pipe to achieve the desired spatiotemporal distribution of temperature, O_2_ and CO_2_ concentration in USIG.

#### 3.1.2 Coefficients in the Multiphysics model of USIGVS

The coefficients in the Multiphysics model of USIGVS (Eq. (1) ∼ Eq. (5)) were specified according to experiments and documents and listed in Table 1 [32,33].where *p*_*0*_ (1.01 10^5^ Pa) is absolute pressure of air. The fourth-order polynomial functions were used to fit relations between *μ*, *v*, *Cp*, *k* and T as follows:

(7) $$\mu \left(T\right)=-8.4∙10^{-7}+8.4∙10^{-8}T-7.7∙10^{-11}T^{2}+4.6∙10^{-14}T^{3}-1.1∙10^{-17}T^{4}$$

(8) $$C_{p}\left(T\right)=1047.7-0.4T+9.5∙10^{-4}T^{2}-6.0∙10^{-7}T^{3}+1.3∙10^{-10}T^{4}$$

(9) $$k\left(T\right)=-2.2∙10^{-3}+1.2∙10^{-4}T-7.9∙10^{-8}T^{2}+4.1∙10^{-11}T^{3}-7.4∙10^{-15}T^{4}$$

(10) $$v\left(T\right)=4.2∙10^{-10}-5.1∙10^{-8}T+2.2∙10^{-6}T^{2}-4.2∙10^{-5}T^{3}+2.9∙10^{-4}T^{4}$$

**Table 1:** Coefficients in the Multiphysics model of USIGVS.(6)$\rho \left(T\right)=0.0035\;p_{0}T^{-1}$

coefficients	value	unit	significance
*ρ*	*ρ*(T)	Kg m^−3^	Air density
*μ*	*μ*(T)	Pa s	Air viscosity
*v*	*v*(T)	m^3^ s^−1^	O_2_ consumption rate
*C*_*p*_	*C*_*p*_(T)	J kg^−1^ K^−1^	Heat capacity of air
*k*	*k*(T)	W m^−1^ K^−1^	Thermal conductivity of air
*D*_*o*_	1.9·10^−5^	m^2^ s^−1^	Diffusion coefficient of O_2_
*D*_*c*_	1.4·10^−5^	m^2^ s^−1^	Diffusion coefficient of CO_2_
*H*	1.56·10^7^	J kg^−1^	Respiratory enthalpy of human
*R*	0.98	dimensionless	Respiratory quotient of human

As illustrated in Table .1, the *ρ*, *μ*, *v*, *Cp* and *k* is the function of temperature varying from 15 °C to 40 °C. Based on experimental data, the relationship between *ρ* and *T* can be written as.

According to Multiphysics model of USIGUV (Eq. (1) ∼ Eq. (10)), the air flow rate (**w**), temperature (*T*), O_2_ concentration [*O*_*2*_] and CO_2_ concentration [*CO*_*2*_] in USIG are strongly coupled with each other through variables and parameters, which makes it possible to only use one actuator of the valve of left inlet pipe (Fig. 1) for regulation of both temperature and gases concentration.

#### 3.1.3 Boundary conditions

Based on the practical operation of the closed-loop USIGVS control system, the boundary conditions for the air flow rate, heat, O_2_ and CO_2_ transport were listed in Table 2.

**Table 2:** Boundary conditions of extended Multiphysics model of closed-loop USIGVS control system.

Boundary	Air flow rate	Temperature	O_2_	CO_2_
Controlled inlet ①	**#w** = **PID**(*e*(*t*))	**#** -**n**∙(*-k*▽*T*) = *k*(T_ext_ -T);	**#**[o_2_] =	**#**[co_2_]
		*T = T_ext_ = 18	[o_2_]_left_inlet_ = 0.21	= [co_2_]_left_inlet_ = 0.03
Upper inlet ②	**#w** = -**v**_in_top_ = 0.2 m s^−1^	**#** -**n**∙(*-k*▽*T*) = *k*(T_ext_ -T);	**#**[o_2_]	**#**[co_2_]
		*T = T_ext_ = 18	= [o_2_]_top_inlet_ = 0.21	= [co_2_]_top_inlet_ = 0.03
Outlet ③	*p* = 0	-**n**∙(*-k*▽*T*) = 0	-**n**∙(*D*_*o*_▽[o_2_]) = 0	-**n**∙(*D*_*c*_▽[co_2_]) = 0
Inlet pipe sections	**n ∙ w** = **0**	**n ∙ w** = **0**	**n ∙ w** = **0**	**n ∙ w** = **0**
Walls	**w = 0**	**w = 0**	**w = 0**	**w = 0**

In Table 2, the symbol **#** and * represented *Neumann* and *Dirichlet* boundary condition, respectively. The *T*_ext_ was external temperature (18 °C) outside of USIG.

### 3.2 Definition of DRF

As temperature, O_2_ and CO_2_ concentration soared over a critical point along with the ping-pong game carrying on, this situation would be instantaneously detected and measured by the sensors installed at outlet pipe, and fed back to the PID controller, and control signals was immediately generated to actuate the inlet valve, increasing air inflow rate in left inlet pipe to make temperature, O_2_ and CO_2_ concentration come back to their prescribed levels with satisfied dynamic performance characteristics.

Because the DRF should embody multi-objective goals attainment, it is necessarily to combine dynamic response of temperature, O_2_ and CO_2_ concentration into one DRF curve based on their control priority as follows:

(11) $$f\left(t\right)=\alpha_{T}T\left(t\right)+\alpha_{o}\left[O_{2}\right]\left(t\right)+\alpha_{c}\left[CO_{2}\right]\left(t\right)$$

where *α*_*T*_,*α*_*o*_ and *α*_*c*_ represented the weight of temperature, O_2_ and CO_2_ concentration in control objective. The expert grading method was applied for prioritization and weights estimation, obtaining *α*_*T*_ = 0.3,*α*_*o*_ = 0.5 and *α*_*c*_ = 0.2, indicating the control priority was O_2_ > temperature > CO_2_.

A custom reference DRF with ideal dynamic (steady-state and transient) response characteristics to reference step input was upfront formulated, i.e., steady-state proximate to the nominal state and transient response performance with desired characteristics, such as proper delay time, rise time, peak time, overshoot, and settling time to reference step input and control actions (Fig. 6), and therefore a cost function (*s*) was formulated based on the difference between reference and actual DRF as follows:

(12) $$s=\int_{0}^{tf}\left[f\left(t\right)-f_{r}\left(t\right)\right]dt$$

where *t*_*f*_ was final time of digital and real-time simulation, the cost function should be minimized to satisfy design requirements forcing the actual DRF to match reference DRF.

### 3.3 GPSA and response optimization based on digital simulation

#### 3.3.1 Simulation model of the closed-loop USIGVS control system

**Fig. 2:**
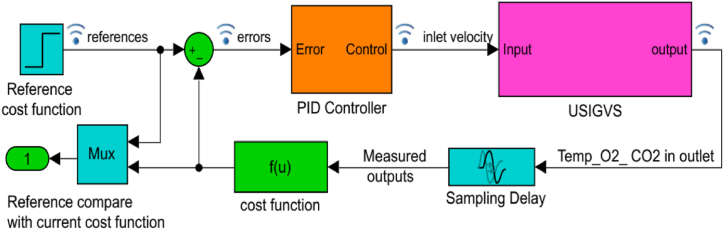
Simulation model of closed-loop USIGVS control system.

According to extended Multiphysics model of the closed-loop USIGVS control system (Eq. (1) ∼ Eq. (5)), the simulation model was established to perform GPSA, response optimization and real-time simulation (Fig. 2).

In the practical application, a sampling delay with 10-s of sensors was set in the simulation model of closed-loop USIGVS control system, which made the air, heat and gases concentrations homogeneous mixing together during the delay time due to convection and diffusion, ensuring the temperature and gases concentrations measured by sensors in outlet pipe were highly in agreement with those in USIG [34,35]. As is known, the larger time-lag might produce dynamic fluctuations causing a negative effect on the response behavior of temperature and gases concentration to control action, nevertheless the 10-s used as sampling delay of sensors was a sufficiently short period of time, compared with the maximum response time of component involved in the closed-loop USIGVS control system, hence 10-s cannot significantly affect the feedback information timeliness and accuracy as well as the closed-loop control effect of USIGVS at all.

#### 3.3.2 Compute-generated random numbers for each parameter

**Fig. 3:**
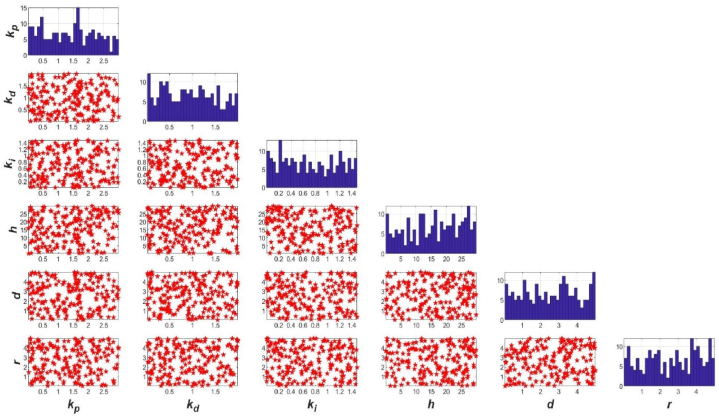
Random numbers of parameter generated through Monte Carlo simulation in design space.

From viewpoint of engineering, the six selected parameters, *h*, *d*, *r*, *k*_*p*_, *k*_*d*_ and *k*_*i*_ were independent from each other, and obeyed uniform random distribution in their respective interval of design space, here 200 random numbers sufficiently representing all possible designs for each parameter were generated through Monte Carlo simulation (Fig. 3).

As illustrated in Fig. 3, the diagonal subplots displayed the histograms of generated parameter values. The off-diagonal subplots were pair-wise scatter plots of the parameters. After the six parameters with different values generated in their respective design space through Monte Carlo simulation, and then they were plugged into extended Multiphysics model of the closed-loop USIGVS control system (Eq. (1) ∼ Eq. (5)), the corresponding 200 random numbers of cost function in 6D space were obtained from digital simulation and Eq. (12). Before digital simulation, the simulation options (Table 3) have been properly set according to the complexity of the problem, and accuracy, speed and the computational expense of the extended Multiphysics model [36,37].

**Table 3:** Settings for extended Multiphysics simulation.

Options	Value	Description
Number of free triangular elements	10^6^	Relatively less finite element number to attain higher precision and faster convergence speed in simulation process.
Element size range of entire geometry	[4.08, 8.15]	Size range of free triangular elements in all domains.
Element size range of boundary	[3.04, 1.56]	Finer grids used on the certain boundaries in Fig. 1 to get more precise solution.
Solver	SPOOLES	Sparse object-oriented linear equations solver applied for calculation and simulation.
Relative tolerance	10^–3^	Solver iterates until this option specified by the corresponding operation feature is fulfilled rather than performs a fixed number of iterations.
Nonlinear method	Automatic (*Newton*)	Use an affine invariant form of the damped *Newton* method repeating the damping-factor reduction until the relative error is less than in the previous iteration.
Minimum damping factor	10^–7^	Minimum value of damping factor for the damped *Newton* method.
Maximum number of iterations	50	Maximum number of iterations allowed performing a discrete solution.
Adaptive mesh refinement	Yes	A method of improving solution accuracy by adapting the mesh to the problem's physical behavior.
Time interval of simulation	24	Simulation lasts 24 h

**Fig. 4:**
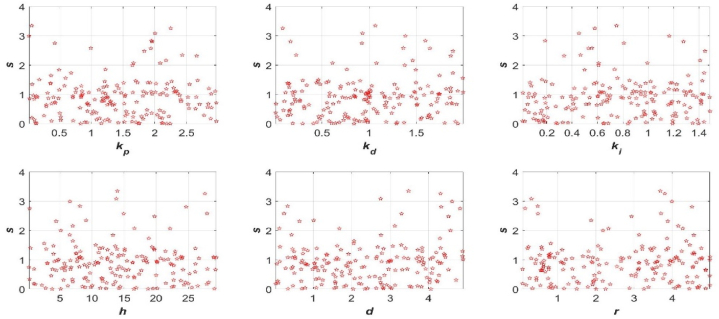
Pair-wise scatter plot of cost function against each parameter.

The 2D projection of the scatter plot of the 6D cost function against each parameter was drawn and illustrated in Fig. 4.

From scatter plots, a minimal value of cost function and corresponding six parameters can be selected from these 200 points in design space of parameters, i.e., *h =* 3.85 m, *d =* 3.12 m, *r =* 1.56 m, *k*_*p*_ = 2.32 m^4^ mol^−1^ s^−1^, *k*_*d*_ = 1.26 m^4^ mol^−1^ and *k*_*i*_ = 0.37 m^4^ mol^−1^ s^−2^, and the corresponding minimal value of cost function *s* = 0.74 was obtained numerically.

#### 3.3.3 GPSA and response optimization for parameters determination

**Fig. 5:**
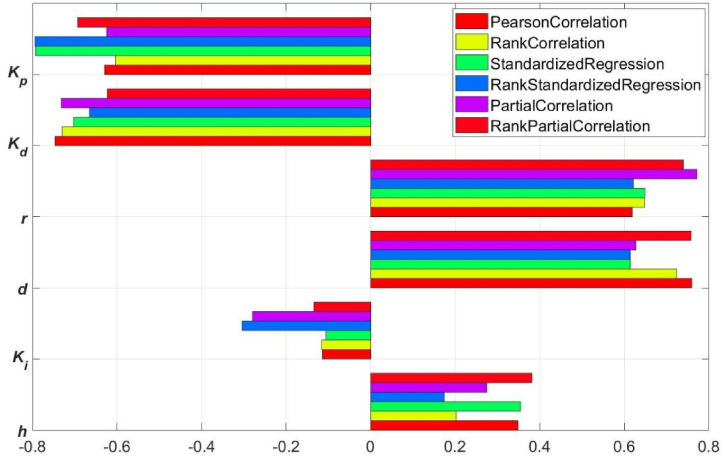
Tornado plot of GPSA performed by different correlation coefficients.

The six coefficients of correlation including *Pearson* correlation, rank correlation, standardized regression, rank standardized regression, partial correlation and rank partial correlation between *h*, *d*, *r*, *k*_*p*_, *k*_*d*_, *k*_*i*_ and *s* was calculated respectively by use of their random numbers. The tornado plot showed the influence of each parameter on the cost function, and the coefficients are plotted in order of influence of parameters on the cost function from top to bottom, the bars are ordered so that they decrease in influence as they go down (Fig. 5).

**Fig. 6:**
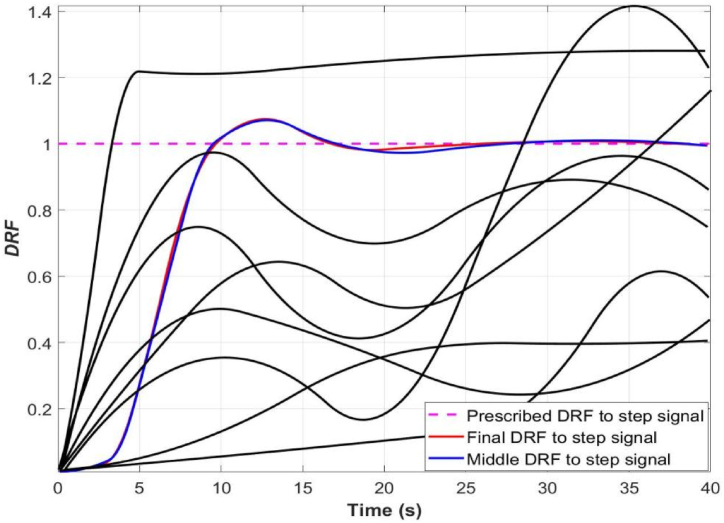
Response optimization of the cost function of closed-loop USIGVS control system.

As illustrated in Fig. 5, the ordering of sensitivity of the cost function to each parameter was generally *k*_*p*_ > *k*_*d*_ > *r* > *d* > *k*_*i*_ > *h*, hence *k*_*p*_, *k*_*d*_ and *r* was the parameter influencing the cost function the most. Based on GPSA, it was an ideal design choice to further optimize *k*_*p*_, *k*_*d*_ and *r* to obtain more optimal cost function signifying more desired dynamic response characteristics of the closed-loop USIGVS control system. Therefore, *k*_*p*_ = 2.32 m^4^ mol^−1^ s^−1^, *k*_*d*_ = 1.26 m^4^ mol^−1^ and *r* = 1.56 m was taken as initial values for further response optimization of cost function based on simulation model of the closed-loop USIGVS control system (Fig. 2). The processes of response optimizations displayed iteratively and converged eventually after running the digital simulation (Fig. 6), and the yielded parameters resulted in the ultimate dynamic responses of DRF (the blue lines) to step signal laid very close to the custom reference DRF curves (the red line) to step signal.

According to response optimization of the cost function of closed-loop USIGVS control system, the more optimal value *k*_*p*_ = 3.17 m^4^ mol^−1^ s^−1^, *k*_*d*_ = 1.49 m^4^ mol^−1^ and *r =* 2.04 m and cost function *s* = 0.40 were obtained and applied for model-based design, building and optimization of the prototype of closed-loop USIGVS control system through real-time simulation.

### 3.4 Model-based design, establishment and operation of closed-loop USIGVS control system prototype based on real-time simulation

After the optimal geometric and tunable parameters of the closed-loop USIGVS control system obtained from GPSA and response optimization through digital simulation (Fig. 2), they were put into engineering application for design, establishment and operation of closed-loop USIGVS control system prototype to practically regulate and control temperature and gases concentration by real-time simulation as follows:

The kinetic model of USIGVS of ping-pong USIG was replaced by its prototype with optimal parameters, *h =* 3.85 m, *d =* 3.12 m, *r =* 2.04 m and the kinetic model of PID controller was substituted by its prototype with optimal parameters, *k*_*p*_ = 3.17 m^4^ mol^−1^ s^−1^, *k*_*d*_ = 1.49 m^4^ mol^−1^ and *k*_*i*_ = 0.37 m^4^ mol^−1^ s^−2^ to form a hardware-in-loop (HIL) structure to carry out real-time simulation on the platform of MatLab/Real-Time Workshop (RTW), implementing prototype design, building and operation of the closed-loop USIGVS control system.

Besides a high-performance computer running the executable *C* code of PID controller generated by rapid prototyping, the HIL included other peripheral equipment such as temperature, O_2_ and CO_2_ sensors, digital amplifier, data acquisition board (NI PCI6221 37-pin) and physical actuators of inlet valve, and so forth. Measurement of temperature, O_2_ and CO_2_ concentration at outlet pipe was made every 10-s, which was a reasonably short sampling time for the closed-loop control of all dynamic processes in the DRF, and guaranteed the air, heat and gases concentrations could be homogeneously mixing together due to convection and diffusion during the sampling period, ensuring the temperature and gases concentrations measured by sensors in outlet pipe were highly in agreement with those in USIG [38].

**Fig. 7:**
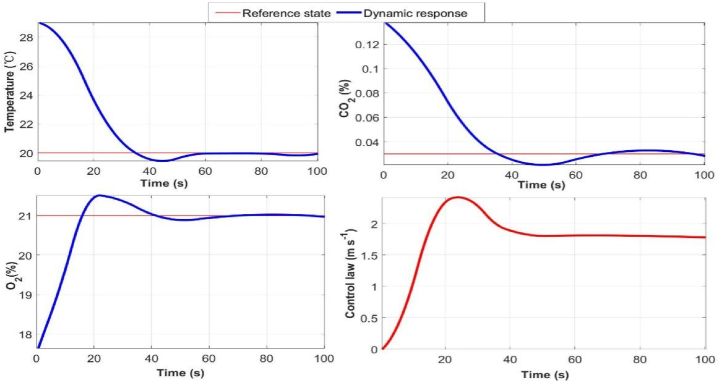
Online control effect of temperature, O_2_ and CO_2_ concentration in USIG through real-time simulation.

The real-time simulation was carried out in ping-pong USIG, the internal variation was caused by respiration and heat release of athletes and audiences, resulting in the temperature, O_2_ and CO_2_ concentration deviated from their nominal levels during the game. The real-time simulation results showed that once the difference between their current values and reference values was detected, measured and fed back into the simulation model of PID closed-loop control law through the temperature and gases concentration sensors and data acquisition board, the control signals generated from the prototype of PID controller were amplified and actuated by inlet valve to regulate the air inflow rate, restoring temperature, O_2_ and CO_2_ concentration in USIG to the prescribed levels with desired transient and steady-state response performance (Fig. 7).

Therefore, the results of real-time simulation clearly verified the effectiveness of the proposed approaches. Furthermore, power consumption was decreased by up to 20.7 % compared with those traditional control and regulation for USIG without model-based prototype design via calculation and analysis.

### 3.5 Discussion

The doors and windows of USIG are opened and closed by spectators constantly in the actual situation, which can be considered as temporary boundary conditions causing internal variations and external disturbances. Although they can temporarily alter the states of temperature, O_2_ and CO_2_ concentration, the feedback control strategy we applied can deal with this problem effectively, because feedback control can effectively to reduce the difference between the output of the closed-loop USIGVS control system and the reference input in the presence of unpredictable disturbances. Based on classic cybernetics, these unpredictable disturbances can always be compensated for within the system. Therefore, the closed-loop USIGVS control system with feedback signals can make the dynamic response characteristics quite insensitive to these temporary boundary conditions in practical application, and therefore these temporary boundary conditions cannot adversely influence closed-loop control effects of the USIGVS.

According to classic cybernetics, the PID controller is often used to control linear system expressed by linear ordinary differential equation and transfer function, the tunable parameters in PID controller is usually determined by root locus in time domain or Bode diagram in frequency domain [39,40]. However, USIG is a 3D Multiphysics model with uncertainties and nonlinearities, therefore it is impossible to specify these tunable parameters by traditional methods, the approaches put forward in the research are good alternative ways to solve the problem. In further research, more information in USIGVS was required to be defined and measured as feedback signals in designing more sophisticated control algorithm, such as nonlinear robust control, machine learning, dissipative structure, and even artificial intelligence, to nonlinearly compensate the system deficient in structure and function, in order to keep it operate steady and reliably according to prescribed design requirements.

## 4 Conclusions

In the article, a prototype of close-loop USIGVS control system for feedback control of temperature and gases concentration in ping-pong USIG was constructed from model-based design, building and operation through digital and real-time simulation. The on-line regulation and control effects showed that the close-loop USIGVS control system with the optimal parameters obtained by GPSA and response optimization can effectively maintain the temperature and gases concentrations at prescribed levels with desired dynamic response characteristics and lower power consumption.

To our knowledge, our paper is first time to carry out the model-based prototype design, building and operation of the closed-loop USIGVS control system obtained directly from highly valid extended Multiphysics model, cybernetics, GPSA and response optimization based on both digital and real-time simulation in a systematic and holistic mode, compared with traditional methods on the basis of expertise, experience, trial-and-error experiments, the methods we applied can greatly increase the system validity and reliability, and save time and cost in construction of USIGVS prototype. This technique will provide a new normal form for design, building and operation of ventilation system for other USIGs, such as basketball, football, volleyball, tennis, badminton, billiards, and so on.

## Data availability

Data will be made available on request. Additionally, other data used and/or analyzed in the current study are available from the corresponding author upon reasonable request.

## CRediT authorship contribution statement

**Ran Meng:** Writing – original draft, Data curation. **Hui Li:** Software, Formal analysis. **Zhiyong Zhang:** Resources, Methodology. **Chen Dong:** Writing – review & editing, Supervision, Project administration.

## Declaration of competing interest

The authors declare that they have no known competing financial interests or personal relationships that could have appeared to influence the work reported in this paper.
